# The future of coffee and cocoa agroforestry in a warmer Mesoamerica

**DOI:** 10.1038/s41598-019-45491-7

**Published:** 2019-06-20

**Authors:** Kauê de Sousa, Maarten van Zonneveld, Milena Holmgren, Roeland Kindt, Jenny C. Ordoñez

**Affiliations:** 1 Department of Agricultural Sciences, Inland Norway University of Applied Sciences, 2322 Hamar, Norway; 2 Bioversity International, 30501 Turrialba, Costa Rica; 3 World Vegetable Center, 741, Shanhua, Taiwan; 4 Resource Ecology Group, Wageningen University, 6708 Wageningen, The Netherlands; 5 World Agroforestry Centre, 30677 Nairobi, Kenya; 6 Latin America Regional Office, World Agroforestry Centre, 1558 Lima, Peru; 7 Facultad de Ingenieria Agroindustrial, Universidad de las Américas, 170125 Quito, Ecuador

**Keywords:** Agroecology, Climate-change ecology

## Abstract

Climate change threatens coffee production and the livelihoods of thousands of families in Mesoamerica that depend on it. Replacing coffee with cocoa and integrating trees in combined agroforestry systems to ameliorate abiotic stress are among the proposed alternatives to overcome this challenge. These two alternatives do not consider the vulnerability of cocoa and tree species commonly used in agroforestry plantations to future climate conditions. We assessed the suitability of these alternatives by identifying the potential changes in the distribution of coffee, cocoa and the 100 most common agroforestry trees found in Mesoamerica. Here we show that cocoa could potentially become an alternative in most of coffee vulnerable areas. Agroforestry with currently preferred tree species is highly vulnerable to future climate change. Transforming agroforestry systems by changing tree species composition may be the best approach to adapt most of the coffee and cocoa production areas. Our results stress the urgency for land use planning considering climate change effects and to assess new combinations of agroforestry species in coffee and cocoa plantations in Mesoamerica.

## Introduction

Adapting agricultural systems to climate change is particularly challenging for perennial crops that take long before farmers fully benefit from their management decisions. Yet, a sense of urgency has developed among farmers, scientists and policy makers across the tropics as climate warming and extreme weather events compromise the productivity of major perennial crops^1^. In Mesoamerica – the area comprising Panama to central Mexico – the productivity of Arabica coffee (*Coffea arabica* L.) is expected to drastically decline as suitable growing areas shift^2^, and pests and pathogens incidence increases under unfavourable climate conditions^3,4^.

Since the first reports of potential impacts of climate change on coffee suitability^2^ an ever growing number of news and blogs from private sector, NGO’s and research organisations are reporting the replacement of coffee by cocoa in zones under 600 m a.s.l. (above the sea level) mainly in Mesoamerica (supplementary information Table S1). According to these sources the drivers of this shift are trends in recent years of increasing coffee production costs and large loses due to pests and diseases (leaf rust crisis)^4^ at low altitudes, attributed to climate change and fuelled by differences in coffee and cocoa prices. All in all, replacing coffee by cocoa has become one of the main strategies for climate change adaptation for producers in low elevation areas^5^, already taking place in Nicaragua, Honduras and El Salvador. Moreover, this strategy is strongly advocated by large NGO’s and development agencies active across the region, under the assumption that areas not suitable for coffee can become unequivocally suitable for cocoa^6^. Nevertheless, there is no quantitative assessment of the feasibility of such strategy, starting from considering that cocoa is vulnerable to climate change itself^7,8^, plus other limitations for transformation of cropping systems.

On the other hand, agroforestry – the deliberate and simultaneous management of trees within crop or livestock systems^9,10^ –, is considered another key strategy to increase the resilience of agricultural systems to climate change^11–13^. Currently, most coffee and cocoa production in Mesoamerica occurs in agroforestry systems^14,15^. Under proper management, agroforestry trees can improve microclimatic conditions that reduce abiotic stress and facilitate the performance of understory crops^16,17^. In addition, farmers can benefit from agroforestry systems by its capacity to provide a number of ecological services, such as water and soil conservation, maintenance of soil fertility and biodiversity conservation^18^. Nevertheless, climate change can also affect the future ecological niches of several tree species^19,20^ and may restrain the prospects of agroforestry as a viable approach for climate adaptation.

To evaluate these two alternatives, shifting coffee-cocoa plantations or maintaining and promoting crops-agroforestry, we assessed the vulnerability of both coffee and cocoa under climate change and the potential impacts of climate change on the habitat suitability for 100 of the most common tree species in coffee and cocoa plantations across Mesoamerica. We modelled current and future climatic niches with ensemble modelling algorithms^21^ using bioclimatic information^22^, downscaled from 17 General Circulation Models, under two Representative Concentration Pathways scenarios of climate change^23^. We selected the intermediate scenario RCP 4.5, which predicts an average temperature increase of 1.4 °C (0.9–2.0 °C), and a scenario with high emissions RCP 8.5, which predicts an average temperature increase of 2.0 °C (1.4–2.6 °C) by 2050 (period 2046–2065). We focus on climate projections for the 2050 s to align with the United Nations framework of global challenges in agriculture and food security^13^. For simplicity, we focus the results in the intermediate scenario and included the variation between the two scenarios assessed here into the main text, the full results for climate change scenario with high emissions are available as supplementary information.

## Results

### Coffee is more vulnerable to climate change than cocoa

**Figure 1:**
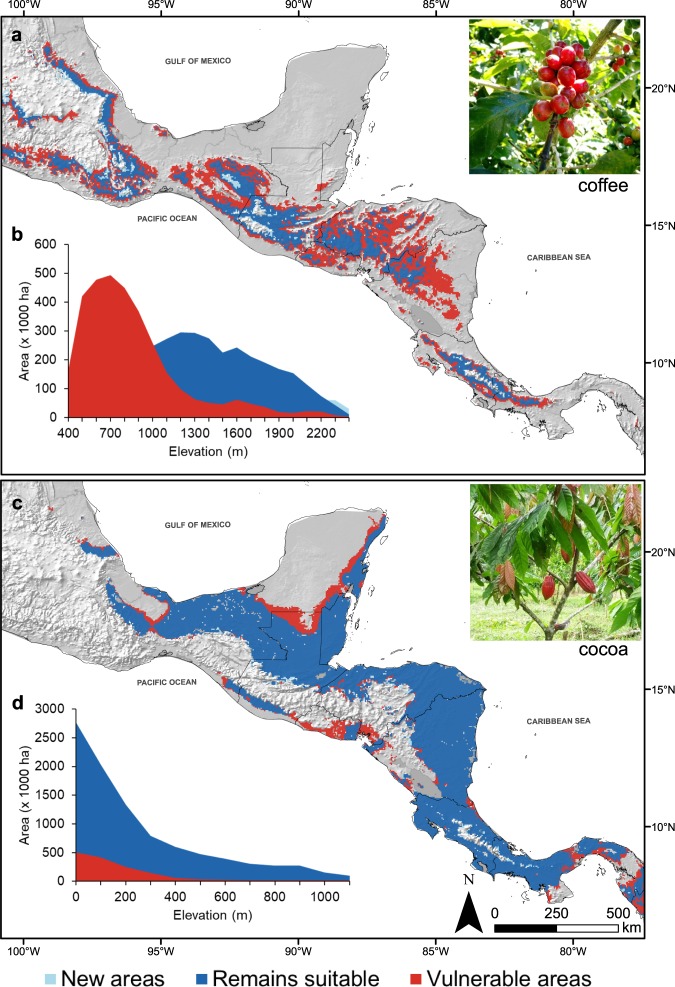
Shifts in suitability due to climate change (RCP 4.5) by 2050 for (**a**) coffee (*Coffea arabica* L.) and (**c**) cocoa (*Theobroma cacao* L.) in Mesoamerica. In (**b**,**d**), shifts in suitability are shown for the altitudinal gradient covered by coffee and cocoa within the continent. Light blue indicate new areas for coffee/cocoa by 2050. Dark blue indicate areas where coffee/cocoa will remain suitable under climate change. Red indicate areas expected to be no longer suitable (vulnerable) for coffee/cocoa under climate change.

Between 55–62% of current areas for coffee production will no longer be suitable by 2050 (Fig. 1a) especially in mid-altitudinal areas (400–700 m a.s.l.). Highlands (>1,800 m a.s.l.) may partly compensate these losses, where coffee will likely expand up to 9–13%. In contrast, cocoa production will probably lose between 13–17% of the current distribution range (Fig. 1a) especially in some lowland areas (0–300 m a.s.l.), expected to become drier in the next decades^19^. Our model projections show that 83–87% of current cocoa areas will remain suitable, especially in the humid areas along the Atlantic coast (0–300 m a.s.l.) (Fig. 1b; Supplementary Fig. S1, Text S1).

**Figure 2:**
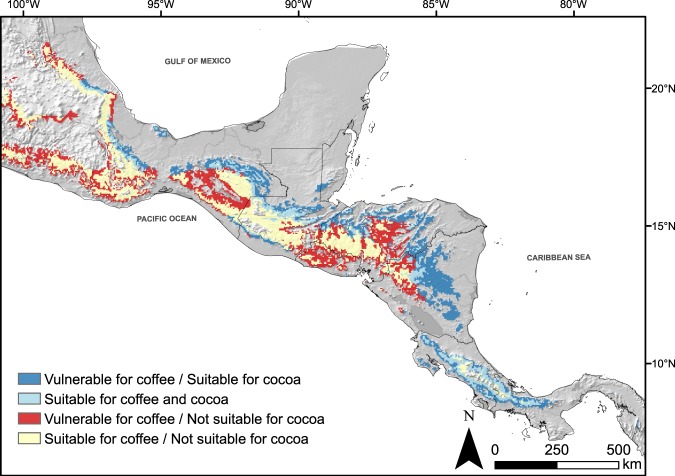
Potential areas in Mesoamerica where cocoa (*Theobroma cacao* L.) can replace coffee (Coffea arabica L.) under climate change (RCP 4.5). Dark blue indicate vulnerable areas for coffee that can be replaced by cocoa. Light blue indicate areas suitable for coffee and cocoa. Red indicate vulnerable areas for coffee where cocoa is not an alternative under climate change. Light yellow indicate remaining areas for coffee where cocoa is not suitable.

Cocoa could potentially replace 85% of the vulnerable coffee areas under climate change in moist regions at elevations under 400 m a.s.l. and 53% at elevations between 400–700 m a.s.l. Areas to be replaced decrease sharply with altitude with no possibility beyond 1,200 m.a.s.l under RCP 4.5 and 1,600 m.a.s.l under RCP 8.5 (Fig. 2, Supplementary Fig. S2).

### Agroforestry trees: winners and losers

**Figure 3:**
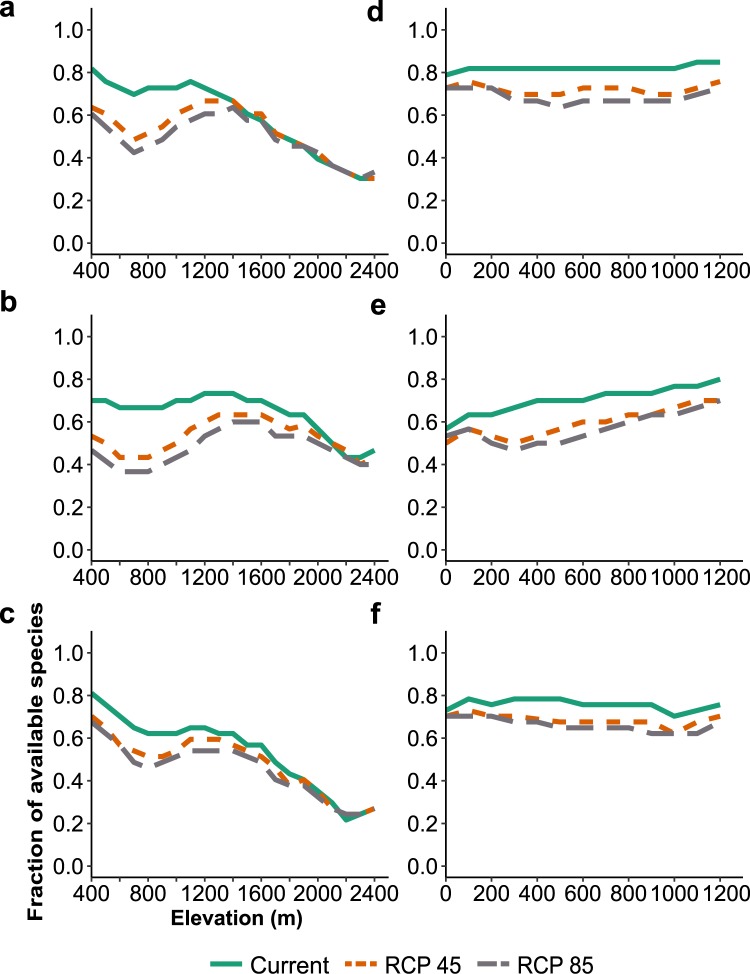
Changes in suitability of the 100 most common tree species in coffee (*Coffea arabica* L.) and cocoa (*Theobroma cacao* L.) agroforestry over the altitudinal gradient in Mesoamerica. Panels a, b and c shows the shifts for fruit, N-fixing and timber trees in coffee areas, respectively. Panels d, e and f shows the shifts for fruit, N-fixing and timber trees in cocoa areas, respectively.

The distribution range of 79% of the tree species assessed in coffee areas and 62% of the tree species assessed in cocoa areas will drastically shrink or become unsuitable in both remaining and vulnerable areas for coffee and cocoa. Major losses are expected for the most popular trees used for fruits, *N*-fixing and timber in mid-altitudinal coffee areas (400–700 m a.s.l.) and lowland cocoa areas (0–300 m a.s.l.; Fig. 3).

**Figure 4:**
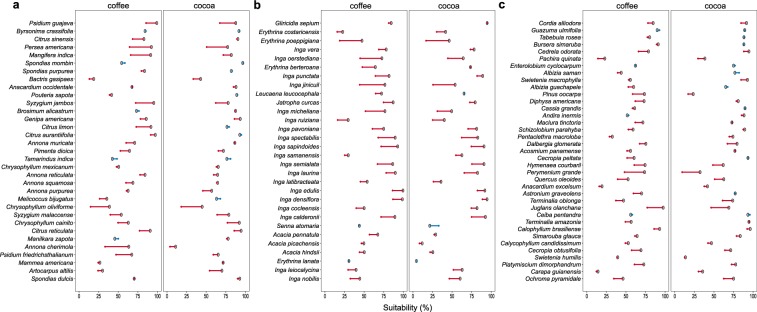
Expected changes in suitability due to climate change (RCP 4.5; expressed as % of current suitable areas) of the most common **a** fruit trees, **b** N-fixing trees and **c** timber trees in coffee (*Coffea arabica* L.) and cocoa (*Theobroma cacao* L.) plantations in Mesoamerica. Grey dot represent the area of a given species under the current climate conditions; Red arrows (left direction), represent decrease in suitable areas; Blue arrows (right direction) represent increase in suitable areas. Species ordered by main use and by their abundance (from to top to bottom) in the inventoried coffee and cocoa farms across Mesoamerica.

Looking at specific tree groups by their main use, we estimate that 20 of the 33 fruit trees will lose more than 15% of their current suitability in coffee areas. The same trend is observed for 14 fruit trees in cocoa suitable areas. The common fruit trees in coffee and cocoa plantations, *Persea americana* (avocado), *Psidium guajava* (guava) and *Manguifera indica* (mango) are among the most vulnerable species with average loss of 53% in suitable areas. Major gains (>15%), however, are found for species such as *Spondias mombin* (jobo) and *Manilkara zapota* (sapodilla) in coffee, *Melicoccus bijugatus* (mamon) in cocoa and *Tamarindus indica* (tamarind) in both coffee and cocoa areas (Fig. 4a, Supplementary Fig. S3).

High losses (>15%) are expected for 25 of the 30 *N*-fixing tree species assessed in coffee and for 18 *N*-fixing tree species in cocoa areas (Fig. 4b, Supplementary Fig. S4). Most common *N*-fixing trees currently growing in coffee and cocoa plantations, such as *Erythrina poeppigiana* (poró), *Inga oerstediana*, *I*. *ruiziana* and *I*. *jinicuil* (guama) are the most vulnerable to expected climate change, with losses of 56% in suitable areas. Only two species, of the selected, may expand their suitability in >26% across cocoa areas, *Inga laurina* (guama) and *Senna atomaria* (vainillo), but only up to 4% in future coffee areas.

In the case of timber trees, we estimate loses of >15% for 22 of 37 species in coffee and 12 tree species in cocoa areas. The most vulnerable timber species include the widely common *Cedrela odorata* (cedar), as well as, the locally important timber species *Perymenium grande* (tatascán) and *Pachira quinata* (pochote), in both coffee and cocoa areas (Fig. 4c, Supplementary Fig. S5). Marginal gains (~5%) are expected for *Albizia saman* (carreto), *Ceiba pentandra* (ceiba) and *Guazuma ulmifolia* (guácimo) in both coffee and cocoa areas.

### Prospects for future coffee and cocoa under agroforestry

Despite the overall losses in suitability for some of the most popular tree species, our projections suggest that agroforestry could persist as a viable alternative to manage coffee and cocoa plantations in Mesoamerica under climate change. By 2050, approximately 72% of coffee areas (both, remaining and vulnerable) will be suitable for more than 30 tree species. This includes a portfolio of at least 10 species per main use (10 fruit species, 10 *N*-fixing species and 10 timber species). Most of these tree species are already present in coffee plantations but mainly in low densities and remain underutilised. Only 9% of coffee areas have very low tree species options (≤3 species).

Our results suggest that cocoa suitable areas have a higher potential for agroforestry than coffee. By 2050, 95% of cocoa areas will be suitable for more than 30 tree species. Only 3% of cocoa areas have very low tree species options (≤3 species) potentially available (Supplementary Fig. S6).

## Discussion

Our results stress the urgency for land use planning that considers potential climate change impacts to define the best areas and growing systems for production of coffee and cocoa under agroforestry management. These results suggest that important changes in tree species composition will be needed for agroforestry systems to remain as the best alternative for climate adaptation of coffee and cocoa fields.

Large areas are highly suitable for cocoa production in Mesoamerica under current climatic conditions and this suitability remains under climate change in 2050, opposing to the trends reported for the current largest cocoa production countries in West Africa^24^. In fact, the total area potentially suitable for cocoa in 2050 in the region could be four times the current world’s cocoa producing area (11 M ha)^25^ stressing the comparative advantage of the region for cocoa production. Despite this large potential, currently Mesoamerica is a minor player in the global cocoa supply chain (providing <1% total world cocoa production in 2017). In general, cocoa production systems in the region include smallholders, with low levels of input use, old plantations and low yields (60–328 kg ha^−1^ year^−1^)^26^. It is argued that this panorama could change substantially if, for instance, farmers used to the management of a specialised perennial crop such as coffee, turn their efforts to cocoa production.

Only considering the coffee vulnerable areas to climate change that will be suitable for cocoa in 2050 (a modest 18% of the total suitable area), there could be 7.5 M ha in Mesoamerica available for cocoa production. Even at the extremely low yields typical of the region, these potentially new producing areas could add 1.5 million tons of cocoa to the global supply. In reality the actual coffee areas that can be replaced by cocoa will be lower than these estimated areas, because farmers may lack financial capacities to transform their coffee plantations^27^ and the capacity to meet the strict existing quality standards. Still, the potential of the region remains large, but fuelling cocoa expansions will require well-structured efforts to i) reduce barriers to transformation, ii) ensure coupling of production to markets and iii) adequate land use planning to avoid expansion of cocoa into natural forests^28,29^ (cocoa suitable areas do coincide with various protected areas within the Mesoamerican Biological Corridor).

Alternatively, by managing agroforestry systems, farmers could potentially maintain their current coffee and cocoa plantations using suitable trees to ameliorate microclimatic conditions. This alternative could also prevent the expansion of agricultural activities towards protected areas that are reported to be suitable in the future^30^. However, it seems highly probable that current agroforestry schemes will need to be modified in terms of species composition, since some of the most popular tree species are also vulnerable to future climate. It is particularly concerning the losses in habitat suitability of *N*-fixing trees such as *E*. *poeppigiana* (poró) and the majority of *Inga* species. These species make up the most abundant agroforestry trees in coffee and cocoa plantations in Mesoamerica^31,32^, and have a key role for the management of soil fertility and sustain more stable productivity^33,34^, especially in low-input and small farming plantations^35^. Therefore, our results anticipate a serious threat for future coffee and cocoa plantations if alternatives for *N*-fixing species are not promptly identified.

Rethinking current agroforestry species composition in coffee and cocoa landscapes requires the identification of the best tree species. Currently, farmers have a clear preference towards few species such as *C*. *odorata* (cedar), *E*. *poeppigiana* (poró), *Inga* spp., *M*. *indica* (mango), *P*. *americana* (avocado) and *P*.*guajava* (guava), all widespread in agricultural fields or open areas and of easy regeneration and propagation. We found that some currently underutilised tree species in coffee and cocoa plantations could potentially maintain or even increase their suitable distribution ranges under future climate, such as the fruit trees *M*. *sapota*, *S*. *dulcis*, *Brosimum alicastrum*, and the timber trees *Simarouba glauca* and *Ceiba pentandra*. These species are present in low densities in coffee and cocoa plantations, and most of them are remnants of previous vegetation^36^.

Expanding the use of underutilised species in agroforestry systems will require a deeper understanding of their agronomic performance considering other factors beyond just climate (e.g. pest, diseases, soil fertility), ecological interactions^37–39^, farmers’ perceptions and local knowledge regarding management and utilisation of these tree species, as well as market incentives to facilitate their wider use. In our assessment, we employed a species distribution modelling (SDM) approach disregarding these aspects. Therefore, the interpretation of our results is driven by the expected changes in biophysical conditions characterised here as changes in extreme precipitation and temperature events. The evidence has shown that these changes are particularly important for agroecosystems in Mesoamerica, and other regions affected by El Niño Southern Oscillation, in which this phenomenon shapes the ecosystem productivity^20,40^, not only across dry regions but also in rainforests^19^.

Here we show that coffee systems are more vulnerable than cocoa systems to climate change. Not only is coffee more sensitive than cocoa to future climate, but also the tree species commonly used in coffee plantations are more vulnerable to the expected climate change. Cocoa as an alternative to coffee could potentially occur in most of the vulnerable coffee areas, but this will require addressing other ecological constraints, the impacts of pest and diseases, costs of technological change and market requirements to determine the real potential of cocoa to replace coffee. Adapting coffee and cocoa to changing climates can benefit from agroforestry systems with a new set of currently underutilised tree species already present in coffee and cocoa plantations. The results of this study are a starting point to develop lines of research that support the re-design of agroforestry schemes and open new venues of research to adapt coffee and cocoa production systems in Mesoamerica.

## Methods

### Selection of tree species

We selected 100 of the most commonly used tree species in cocoa and coffee plantations across Mesoamerica (Supplementary Table S2) using three criteria: (i) abundance assessed from compiled inventories of shade species in smallholder farms across the region^41–43^; (ii) ecological and economic services identified by farmers^44,45^; and, (iii) availability of a minimum of 60 records to ensure accurate modelling results^46^.

From these 100 species, 30 are mainly used due to their potential to improve soil conditions by fixing nitrogen, 37 species mainly used for timber products (within the farm and potentially marketable) and 33 species mainly used as fruit trees^44,45^. The selected species belong to 27 botanical families and most (91 species) are native of the neotropics; the others are economically important species and naturalised fruit trees in Mesoamerica (Supplementary Table S2).

### Compilation and validation of presence location points

We compiled presence location points of selected tree species (including coffee and cocoa) from the Global Biodiversity Information Facility (GBIF)^47^, MAPFORGEN^48^ and from the database of farm inventories used to select the tree species. No distinction was made between locations from natural forests or farms because this information was not always available in the original sources.

Records with no geographic information or with obvious errors such as incomplete coordinates, locations in the ocean and mismatches between administrative data and coordinates were excluded from the analysis. For this, we compared the collected presence data and information on administrative boundaries with information from the DIVA-GIS database^49^, removing the mismatches. Presence locations from 1959 or before were also removed to meet the current baseline climate used. Finally we reduced the possible effects of sampling bias and spatial autocorrelation through systematic sampling^50^. This approach consists in create a grid of a defined cell size (in our case 2.5 arc-min) and randomly sample one presence points per grid cell. In the Fourcade *et al*.^50^ assessment, the approach showed well performance among the other tested approaches irrespective the species and bias type, which is our case.

The final dataset with validated and unbiased presence locations comprised 130,480 occurrences for the 100 tree species combined (Supplementary Table S2), 2,194 location points for coffee and 1,241 location points for cocoa. Since absence locations were not available, for each species, we allocated 1,000 random pseudo-absence locations within the study area, which were sampled (without replacement) using the R^51^ package dismo^52^.

### Climate data

We used bioclimatic predictors (baseline period of ~1960–1990) from WorldClim^22^ at a spatial resolution of 2.5 arc-min. The bioclimatic variables include extreme or limiting factors that are ecologically important based on the variation in precipitation and temperature. We selected the least correlated variables applying an analysis of variance-inflation factors (VIF)^53^, whereby the variables with the highest correlation (VIF > 10) were removed, resulting in nine bioclimatic predictors. Which were: (i) bio02, mean diurnal range; (ii) bio03, isothermality; (iii) bio08, mean temperature of wettest quarter; (iv) bio09, mean temperature of driest quarter, (v) bio13, precipitation of wettest month; (vi) bio14, precipitation of driest month; (vii) bio15, precipitation seasonality; (viii) bio18, precipitation of warmest quarter; and, (ix) bio19, precipitation of coldest quarter.

We based the projections of future distribution in 2050 s on two Representative Concentration Pathways scenarios (RCPs) of climate change from the Intergovernmental Panel on Climate Change (IPCC)^23^. We selected the intermediate scenario RCP 4.5, which predicts an average temperature increase of 1.4 °C (0.9–2.0 °C), and a scenario with very high emissions RCP 8.5, which predicts an average temperature increase of 2.0 °C (1.4–2.6 °C) by 2050 (period 2046–2065). We focus on climate projections for 2050 to align with the United Nations framework of global challenges in agriculture^13^. For each selected scenario, we predicted species suitability using the 17 General Circulation Models (GCM) available for both RCP scenarios (Supplementary Table S3).

### Data analysis

We modelled the distribution of all species within the longitudes −101 and −77, and the latitudes 7 and 22. All analyses were done in R^51^ using a consensus method for species distribution modelling (SDM) compiled by the package BiodiversityR^21^, which calculate ensemble suitability as a weighted average of probabilities predicted by 17 SDM algorithms (Supplementary Table S4). Previous studies have shown that the consensus method based on weighted averages can significantly increase the accuracy of SDM^54^.

For the model calibration, we performed a 4-fold cross-validation by randomly assigning (without replacement) location data to four bins. The performance of different SDM algorithms was evaluated for each bin separately after algorithms were calibrated with data from the other three bins. The SDM performance was assessed by the area under the curve (AUC^55^) criterion computed by the R package PresenceAbsence^56^. Although some authors tend to criticise this method, the evidence^57^ has shown that AUC has strong correlation with the presence-absence threshold that makes sensitivity equal to specificity and remains a valid measure of relative model performance. Considering that, predictions from each of the 17 SDM algorithms were transformed to AUC weights by dividing each by the total of all AUC predictions. We selected the SDM algorithms with AUC weights >0.05, which means at least 5% of contribution to the consensus predictivity^21^, and recalculated weights to sum to one^53^. The AUC values for the selected SDM models are shown in supplementary information Fig. S7.

Therefore, selected SDM algorithms were used to obtain the suitability model for coffee, cocoa and the 100 tree species. We then applied the derived suitability model to each of the 17 downscaled GCMs to predict the distribution of suitability by the 2050 s. For each species, ensemble suitability maps for baseline and future climates were converted in absence-presence maps with the recommended threshold method of maximum sensitivity (true positive) + specificity (true negative)^58,59^.

Since there are no criteria to assess which of the GCMs best predict future climate, by incorporating all 17 GCMs we included all plausible changes in the distribution of the focal species. The results of the 17 GCMs presence-absence layers were integrated into a single layer, using the criterion of likelihood scale^60^, which requires at least 66% of agreement among GCMs to keep the predicted presence or absence in a given grid cell.

Organising the datasets relied on R packages magrittr^61^ and tidyverse^62^. Layers were processed using the R packages maptools^63^, raster^64^, rgeos^65^ and rgdal^66^. To produce Figs 3, 4, S3, S4, S5 and S7, the R packages ggplot2^67^ and svglite^68^ were used.

## Supplementary information

Supplementary Information

## Data Availability

Data and R code used is available through Dataverse^69^. The full project replication workflow is available through GitHub https://github.com/agrobioinfoservices/enm_agroforestry.
