# Supplementary material for: The future of coffee and cocoa agroforestry in a warmer Mesoamerica
Source: Sci Rep. 2019 Jun 20;9:8828. doi: 10.1038/s41598-019-45491-7 (PMC6586680; doi:10.1038/s41598-019-45491-7)
Supplement: Supplementary file 1 — Supplementary Information [file 41598_2019_45491_MOESM1_ESM.pdf]

Supplementary Information for

The future of coffee and cocoa agroforestry in a warmer Mesoamerica

Kauê de Sousa*, Maarten van Zonneveld, Milena Holmgren, Roeland Kindt, Jenny C. Ordoñez

***Corresponding author**
kaue.desousa@inn.no; k.desousa@cgiar.org

This PDF file includes:

Supplementary text
Figs. S1 to S7
Tables S1 to S4

Supporting Information Text

SI Results

Changes in suitability. Coffee plantations in drier ecological zones with annual precipitation lower than 1,400 mm (under the baseline climate) are the most sensitive including the highlands in Mexico across the Pine-Oak Forests, Balsas Dry Forests and Chiapas Depression; midlands in Honduras, in Olancho and Francisco Morazán; Guatemala in Jutiapa, Baja Verapaz and Huehuetenango. Losses are also expected in moist regions along the Atlantic Coast, mainly in the lowlands across the Moist Broadleaf Forests of Matagalpa and Jinotega in Nicaragua. New suitable regions for coffee are identified in the highland Mexico in the Sierra Madre Oriental Pine-Oak Forests and Volcanic Belt Pine-Oak Forests of Oaxaca, Chiapas, Puebla, and Michoacán; in highland Guatemala in the Pine-Oak Forests of Huehuetenango, Quiche and San Marcos; and in highland Costa Rica in the Talamancan Montane Forests of San Jose, Cartago, and Alajuela (Fig. 1a, Fig. S1a).

Suitable areas for cocoa production will likely decrease in 13-17% by the 2050s. New areas with suitable climates for cocoa production will account for 2% of future areas. The most vulnerable areas for cocoa are mainly in lowland Mexico in the Yucatán Moist Forests and the Petén-Veracruz Moist Forests of Quintana Roo, Campeche and Oaxaca; lowland Guatemala in the Petén-Veracruz Moist Forests of Petén; and lowland Panama in the Isthmian-Atlantic Moist Forests in Panama; and in the Dry Forests of lowland El Salvador. The suitability of cocoa under climate change is maintained in 83-87% of its current areas in elevations across the Tropical Moist Broadleaf Forests in Mesoamerica (Fig. 1b, Fig. S1b).

Potential areas for shifting coffee with cocoa comprise Mexico across the moist forests in Sierra De Los Tuxtlas, the Sierra Madre de Chiapas and Petén-Veracruz. Also, the Moist Forests of Nicaragua in Atlantico Sur, Jinotega and Chontales. The Moist Forests and Pine-Oak Forests in Honduras (Olancho, Yoro, Cortes and Santa Barbara). Finally, the Moist Forests and Pine-Oak Forests in Guatemala in Petén-Veracruz, Alta Verapaz, Izabal, Peten, Quiché and Huehuetenango; and the Seasonal Moist Forests in Costa Rica in Puntarenas (Fig. 2, Fig. S2).

Agroforestry trees availability. For coffee, areas with high potential to select tree species from a portfolio of at least 10 species per main use include the highlands across the ecological zones in Mexico in the Pine-Oak Forests and Petén-Veracruz Moist Forests of Chiapas, Guerrero, Oaxaca, Veracruz and Puebla. In Guatemala, areas with high potential include Quiché, Alta Verapaz, Jalapa, Chimaltenango and Huehuetenango. In Honduras, the Pine-Oak Forests and Mountain Forests of Francisco Morazán, Intibuca, Comayagua, La Paz and Olancho. For Nicaragua, we identify high potential in Jinotega, Estelí, Matagalpa and Madriz. In Costa Rica in the Talamancan Montane Forests of San Jose, Heredia, Alajuela, Cartago, and Puntarenas.

Vulnerable areas for coffee by the 2050s (where coffee is expected to lose suitability) with high potential for agroforestry (at least 10 species per main use) cover the midlands in Mexico in the Petén-Veracruz Moist Forests, the Central American Mountain Forests and Pine-Oak Forests of Chiapas, Oaxaca, and Veracruz. In Guatemala, across Alta Verapaz, Izabal, and Suchitepequez. For Nicaragua, across Atlantico Sur. In Costa Rica, across Puntarenas and San Jose. And, the province of Chiriqui in Panama.

Areas with high vulnerability (< 3 species per main use) are identified across the midlands Pine-Oak Forests and Dry Forests of Honduras (Comayagua, Paraíso, Yoro and Lempira), Nicaragua (Nueva Segovia, Jinotega and Madriz), El Salvador (La Libertad and Santa Ana) and highlands in Mexico (Oaxaca and Morelos).

For cocoa, areas with high potential for selecting trees from a portfolio of more than 30 species (10 per main use) cover all the humid tropical forest at lowlands across the Pacific coast in Costa Rica, Atlantic coast in Nicaragua, Mosquitia in Honduras, Belize, lowlands north of Cobán and south of Sierra Madre in Guatemala and lowlands in the Gulf of Mexico. There is also a high potential for selecting more than 30 species across the transition zones of the Central American Moist Forests and Central American dry forests in Nicaragua (Atlantico Sur), Guatemala (Quetzaltenango), the Dry Forests of El Salvador (Ahuachapan, Sonsonate and Usulutan) and the Moist Forests of Costa Rica (Limon). Vulnerable cocoa areas with low agroforestry options (< 3 species), are identified across the Dry Forest of Honduras (Comayagua, Yoro and Nueva Segovia).

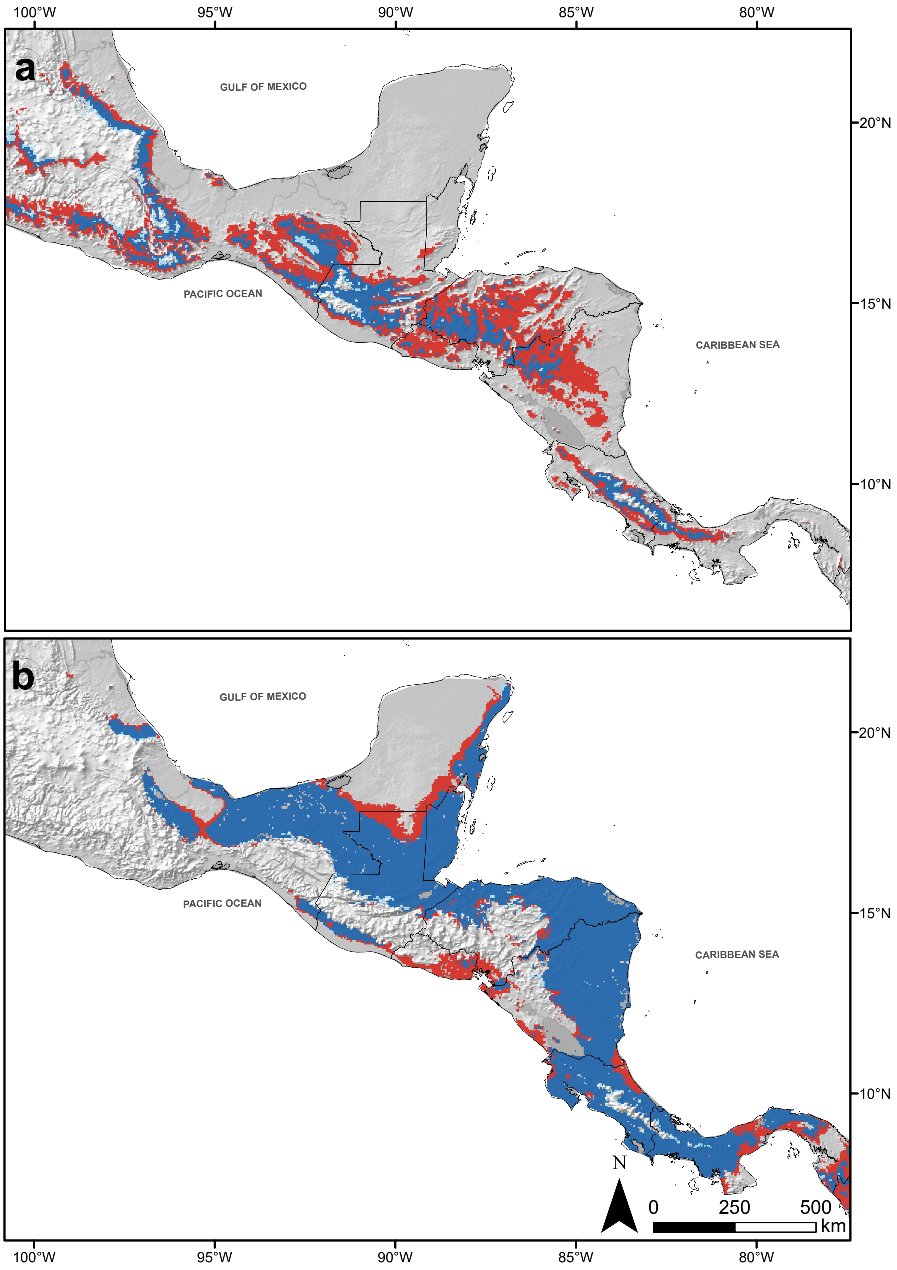

Fig. S1. Shifts in suitability due to climate change (RCP 8.5) by 2050 for **a** coffee (*Coffea arabica* L.) and **b** cocoa (*Theobroma cacao* L.) in Mesoamerica. Light blue indicate new areas for coffee/cocoa by 2050. Dark blue indicate areas where coffee/cocoa will remain suitable under climate change. Red indicate areas expected to be no longer suitable (vulnerable) for coffee/cocoa under climate change.

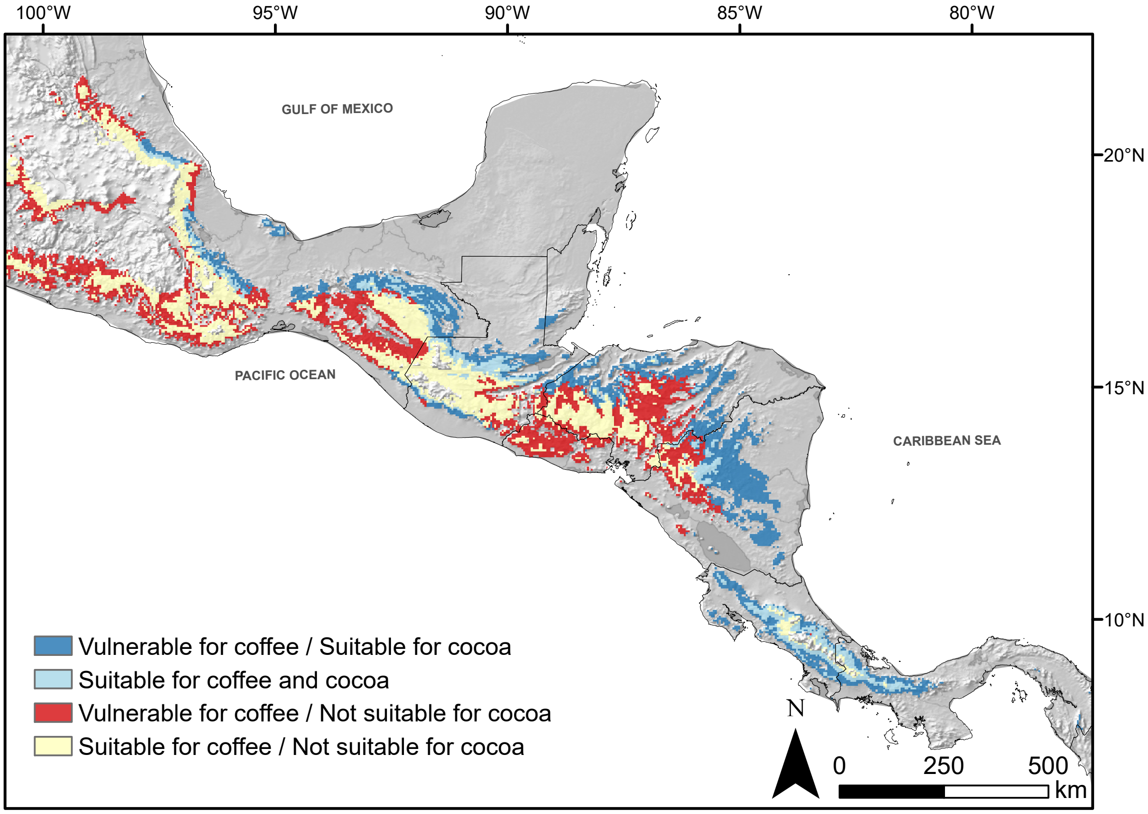

Fig. S2. Potential areas for coffee (*Coffea arabica* L.) shifting using cocoa (*Theobroma cacao* L.) in vulnerable areas for coffee under climate change (RCP 8.5) across Mesoamerica. Dark blue indicate vulnerable areas for coffee that can be replaced by cocoa. Light blue indicate areas suitable for coffee and cocoa. Red indicate vulnerable areas for coffee where cocoa is not an alternative under climate change. Light yellow indicate remaining areas for coffee where cocoa is not an alternative.

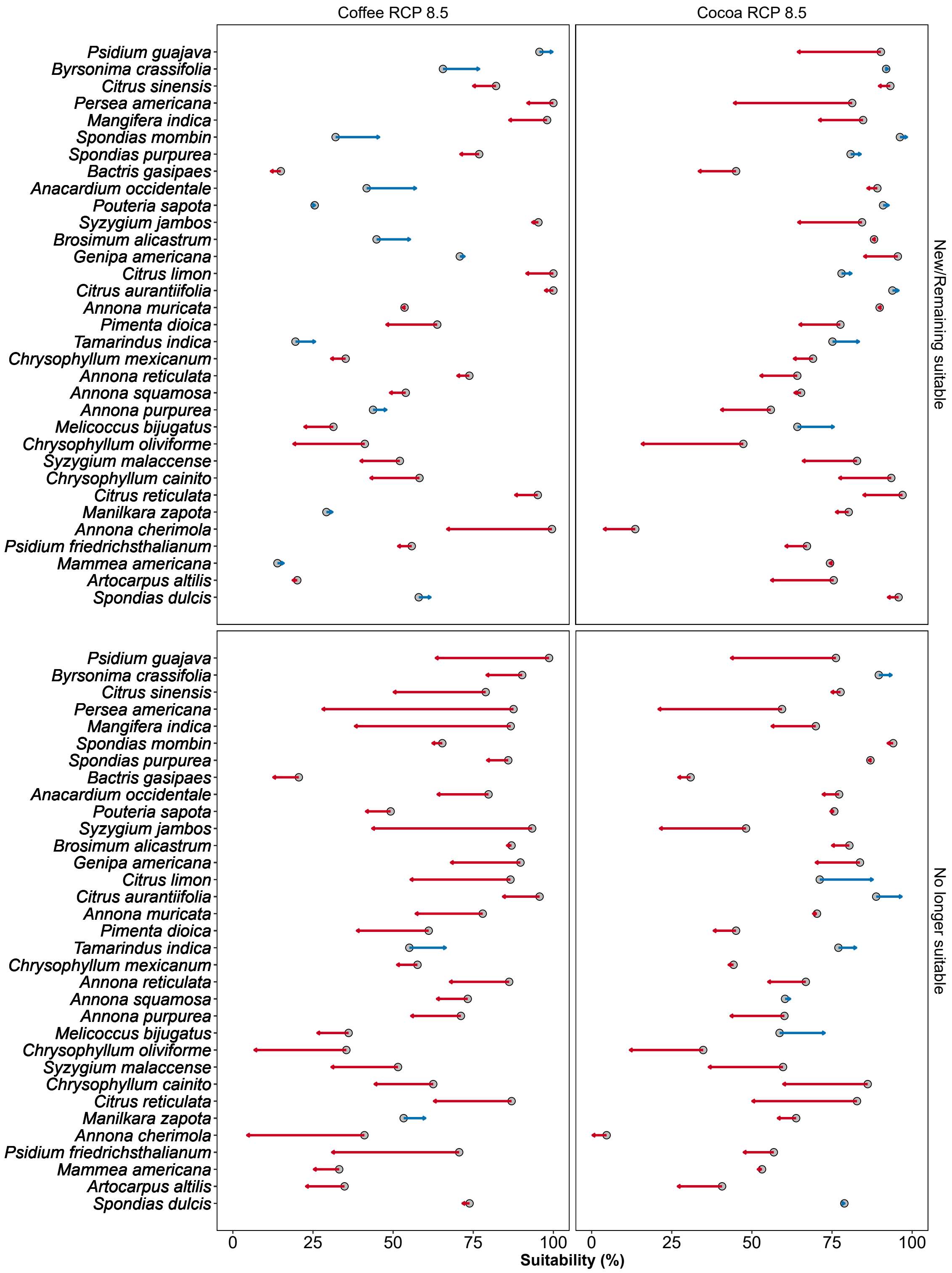

Fig. S3. Expected changes in suitability of fruit tree species (expressed as % of current suitable areas), in new/remaining areas and vulnerable (no longer suitable) areas for coffee (*Coffea arabica* L.) and cocoa (*Theobroma cacao* L.) growing areas under climate change (RCP 8.5). Grey dot represent the distribution of a given species under the current climate conditions; Red arrows (left direction), represent decrease in suitable areas by the 2050s; Green arrows (right direction) represent increase in suitable areas by the 2050s.

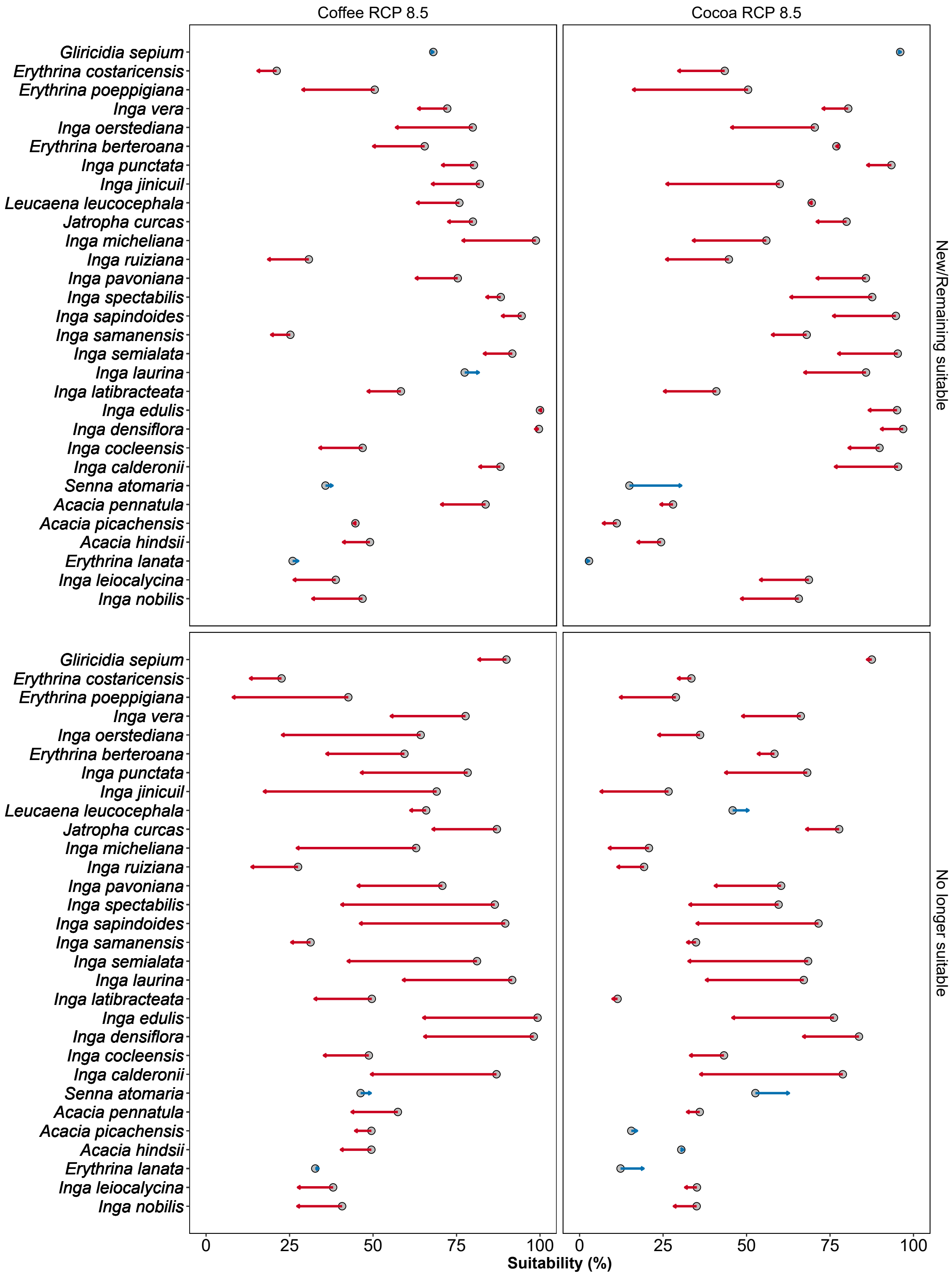

Fig. S4. Expected changes in suitability of N-fixing tree species (expressed as % of current suitable areas), in new/remaining areas and vulnerable (no longer suitable) areas for coffee (*Coffea arabica* L.) and cocoa (*Theobroma cacao* L.) growing areas under climate change (RCP 8.5). Grey dot represent the distribution of a given species under the current climate conditions; Red arrows (left direction), represent decrease in suitable areas by the 2050s; Green arrows (right direction) represent increase in suitable areas by the 2050s.

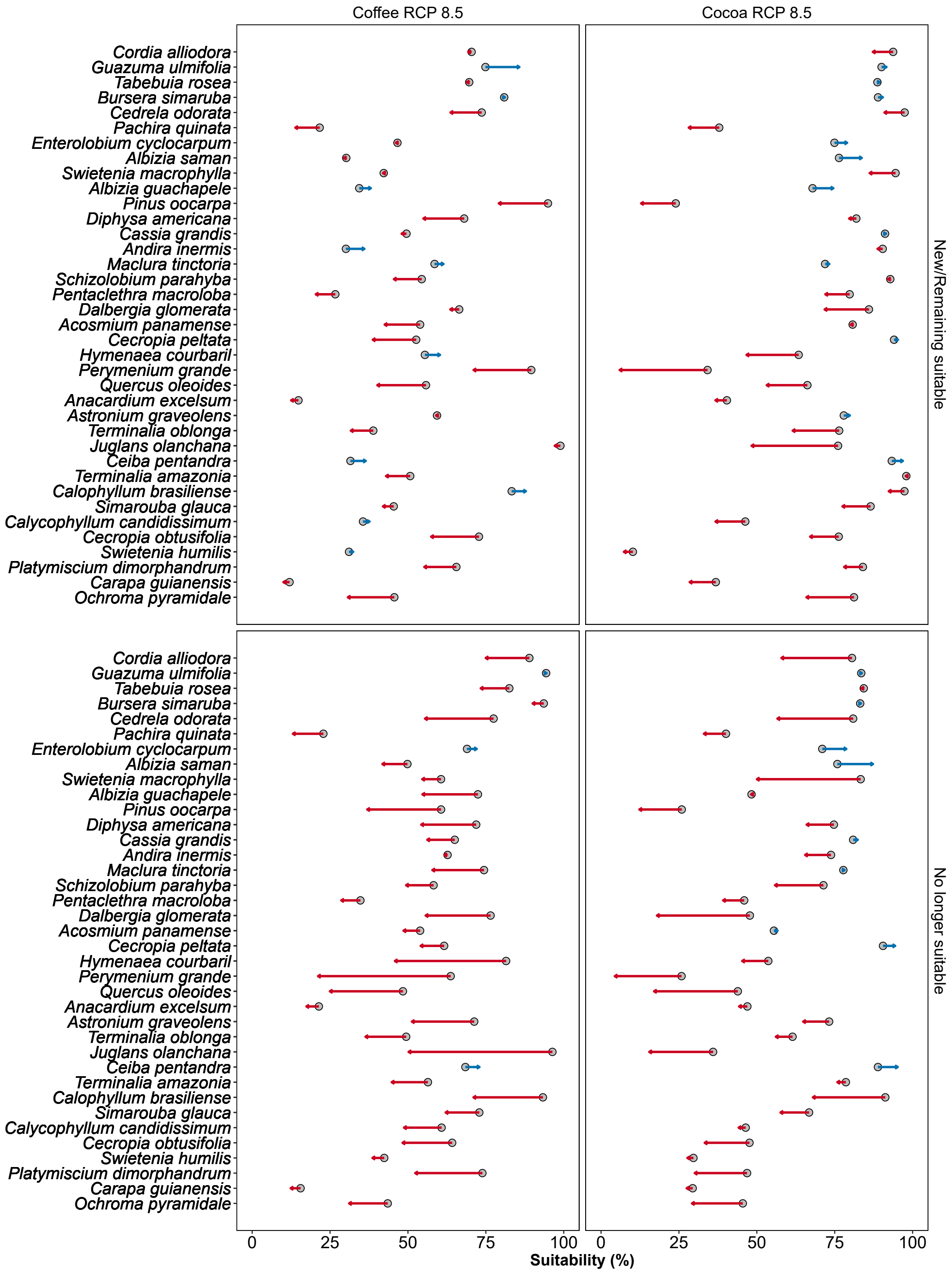

Fig. S5. Expected changes in suitability of timber tree species (expressed as % of current suitable areas), in new/remaining areas and vulnerable (no longer suitable) areas for coffee (*Coffea arabica* L.) and cocoa (*Theobroma cacao* L.) growing areas under climate change (RCP 8.5). Grey dot represent the distribution of a given species under the current climate conditions; Red arrows (left direction), represent decrease in suitable areas by the 2050s; Green arrows (right direction) represent increase in suitable areas by the 2050s.

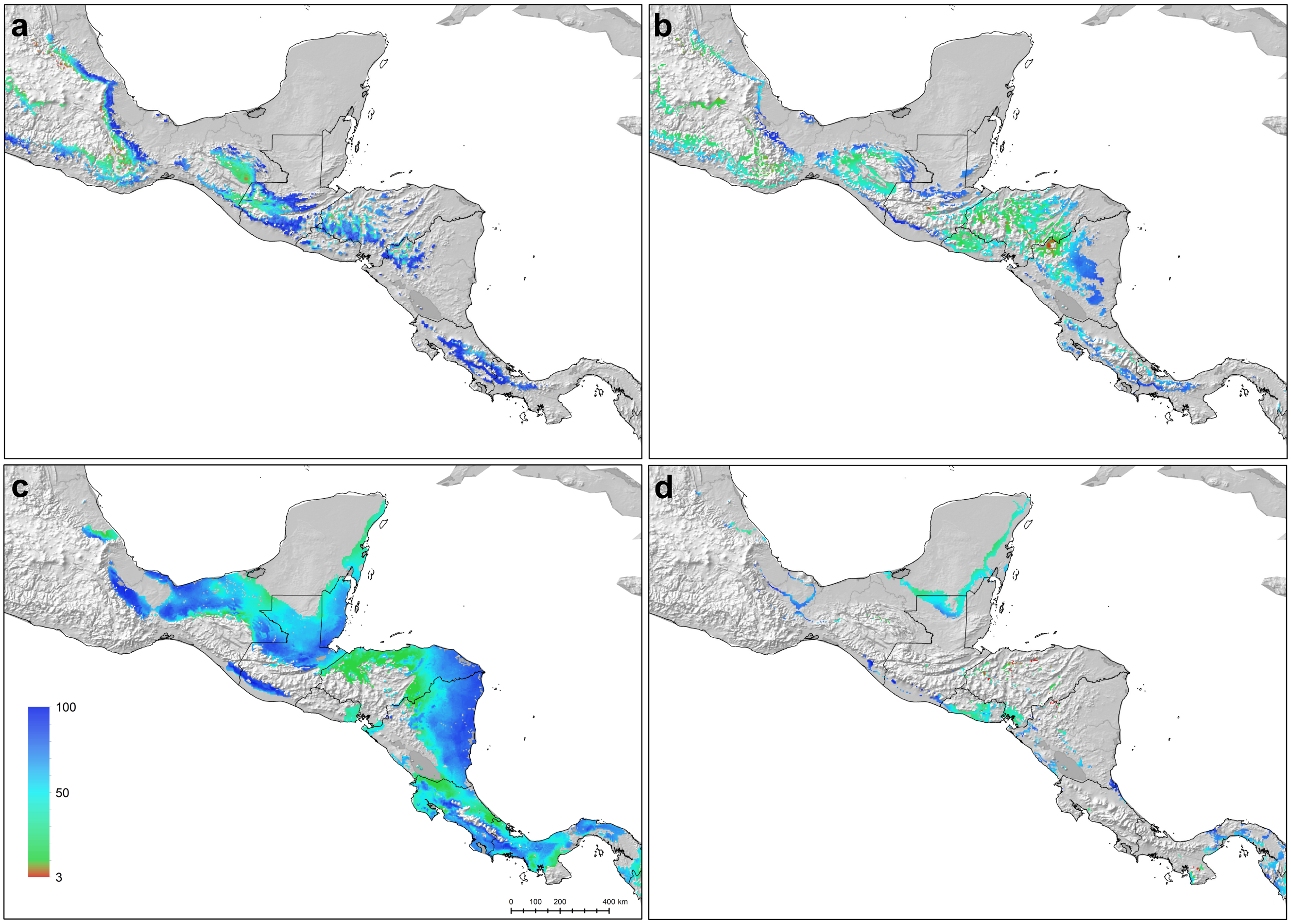

Fig. S6. Distribution of the agroforestry species within **a** suitable and **b** vulnerable (no longer suitable) areas for coffee, and **c** suitable and **d** vulnerable areas for cocoa under climate change (RCP 4.5) across Mesoamerica. Colour gradient, from red to dark blue, represent the number of available species per grid-cell at a resolution of 2.5 arc-min (5 km).

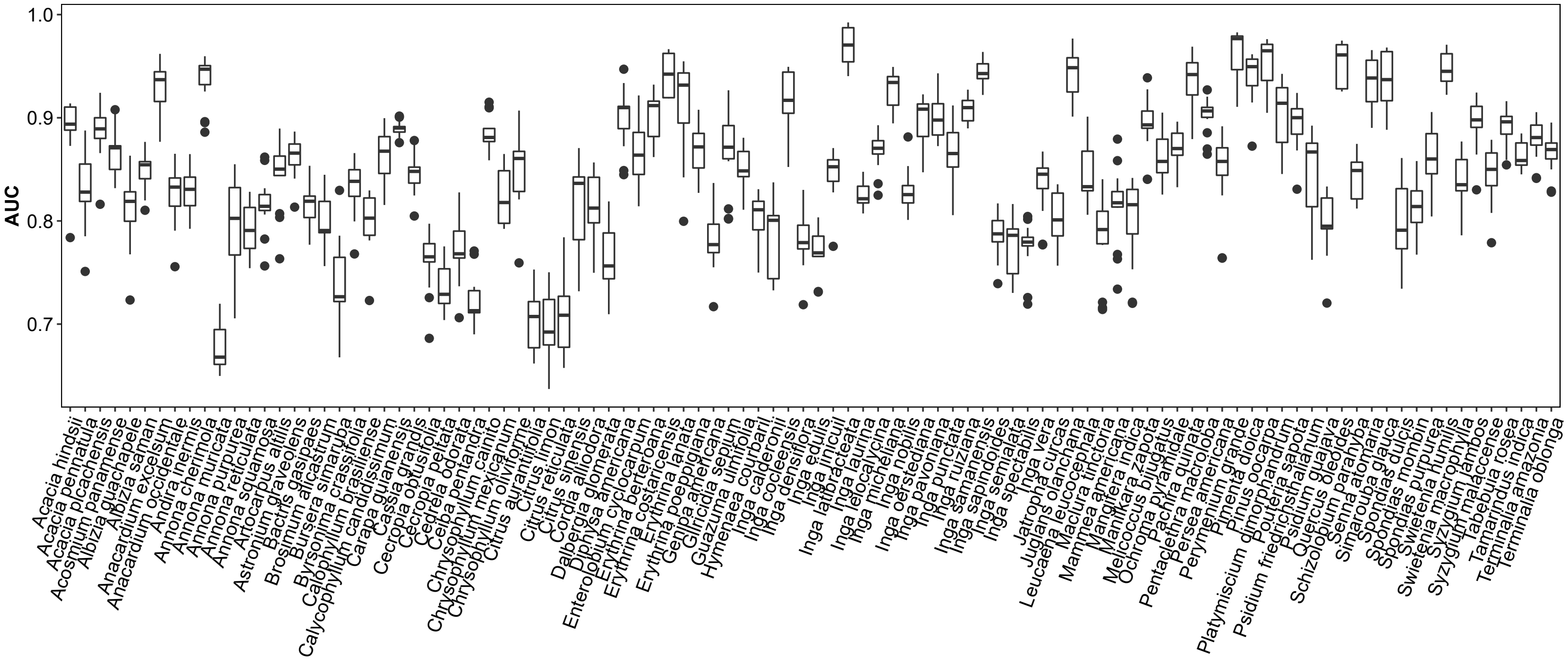

Fig. S7. Box plot of AUC values from SDM algorithms selected for the ensemble model.

Table S1. Summary of news reports about coffee replacement by cocoa in Central America (period 2013-2019).

Organization	Year	Country / Region	Main report	Source
Thomson Reuters Foundation	2016	El Salvador, Nicaragua	Farmers started reintroducing cocoa in 2014, coffee losses in low areas meant people could not make a living anymore.	1
Bangor daily news	2016	Central America	Coffee exports decreased in 2014 and cocoa exports increased in 2015, areas are being abandoned due to leaf rust crisis, low coffee prices combined with high cocoa prices	2
Global Coffee Report	2015	Honduras	Honduras plan to replace 8 per cent of its coffee plantations with cocoa farms in 2016, they want to reach to 60,000 ha.	3
MSN news (video)	2016	Nicaragua	Farmers from low coffee areas are switching to cocoa, due to crop unsuitability and better prices of cocoa.	4
CRS blog	2016	Nicaragua	Company sees trends of small farmers from low lands replacing coffee with cocoa, where costs of production and losses are increasing. The transition is not as fast the company would like due to limitations like cultural binding to coffee production and lack of credit given that investments for cocoa would require 4-5 years before the first harvest.	5
LRS story hub	2018	El Salvador	LRS report a trend of coffee replacement with cocoa, they see it as an option to rescue cocoa producing tradition, with local high value varieties. Cocoa alliance (CRS-LRS) project, provides technical assistance, market information, and genetic material to facilitate the transition and develop the cocoa sector in El Salvador.	6
Medium Corporation	2016	Latin America	Sustainable Harvest's coffee supply chain conference, speaker met cooperatives from Peru and Nicaragua where farmers are already switching coffee for cocoa. There are advantages of replacement, including areas with more suitable conditions, less labor, transitional models to reduce costs of transition, more cocoa products. Disadvantages include risk of drought, small market for high price cocoa and not possibility to compete with Africa for bulk cocoa.	7
CBC Canada	2017	Central America	Coffee crop suffers from climate change, some options to maintain coffee production are discussed as well as the option of coffee replacement, not actual trends	8
LEGISCOMEX	No date	Central America	Financial report about the production increase of fine cocoa in the region. Reports that replacement of coffee is taking place in 3 countries.	9
EL mundo	2015	El Salvador	Reports that already in 2012 farmers from low lands started shifting to cocoa, due to attack of pest and diseases and high coffee loses. Initial investment for both crops is similar and there is no technical advice on how to do the replacement. The governments is also assessing the feasibility of the strategy with key actors of the coffee and cocoa sector.	10
Swiss Cooperation	2018	Nicaragua	Swiss Cooperation is developing a large program to finance the expansion of cocoa, although replacement of coffee is not an objective of the program in itself, it is mentioned as one of the drivers of cocoa expansion in Nicaragua.	11
CATIE Nicaragua	No date	Nicaragua	CATIE reports about the support of one of its large development programs to replacement of coffee by cocoa in farms under 600 m.a.s.l.	12

Sources:
1. https://www.reuters.com/article/us-centralamerica-climatechange-coffee-c-idUSKCN10Z0VX
2. https://bangordailynews.com/2016/01/18/business/central-american-farmers-turn-to-cocoa-as-climate-change-threatens-coffee/
3. http://gcrmag.com/regions/view/honduras-to-replace-coffee-with-cocoa
4. https://www.msn.com/es-mx/noticias/opinion/sweet-dreams-as-coffee-farmers-turn-to-cocoa/vi-BBofZbY
5. https://coffeelands.crs.org/2016/04/knocking-on-coffees-door-cocoas-case-as-a-coffee-farm-alternative/
6. https://lwr.org/story-hub/bean-bar-reviving-cocoa-el-salvador
7. https://medium.com/@katie.gilmer.pon/with-coffee-in-crisis-farmers-look-to-cacao-a2d3a8d285c2
8. https://www.cbc.ca/news/technology/coffee-climate-change-threatens-1.4285388
9. https://www.legiscomex.com/Documentos/centroamerica-busca-mina-oro-cacao-fino-de-aroma-clara-villatoro-actualizacion
10. https://elmundo.sv/crisis-del-cafe-impulsa-propuesta-de-sustituir-el-bajio-con-cacao-fino/
11. https://www.swissinfo.ch/spa/ayuda-al-desarrollo-de-suiza_el-cacao-de-la-esperanza-en-nicaragua/43788304
12. https://www.catie.ac.cr/nicaragua/es/78-cambio-climatico-proponen-cacao-como-alternativa-al-cafe.html

Table S2. Most common tree species in cocoa (*Theobroma cacao* L.) and coffee (*Coffea arabica* L.) systems across Mesoamerica selected for the study. Ordered by family name.

Begin of Table S2					
Family	Species	Frequency	Main use	Neotropical	Presence locations
Anacardiaceae	Mangifera indica	0.8110	Fruit	No	620
Anacardiaceae	Spondias mombin	0.6960	Fruit	Yes	1256
Anacardiaceae	Spondias purpurea	0.6160	Fruit	Yes	634
Anacardiaceae	Anacardium occidentale	0.3300	Fruit	Yes	1247
Anacardiaceae	Anacardium excelsum	0.1890	Timber	Yes	234
Anacardiaceae	Astronium graveolens	0.1820	Timber	Yes	557
Anacardiaceae	Spondias dulcis	0.0101	Fruit	Yes	68
Annonaceae	Annona muricata	0.1590	Fruit	Yes	564
Annonaceae	Annona reticulata	0.0640	Fruit	Yes	688
Annonaceae	Annona purpurea	0.0600	Fruit	Yes	238
Annonaceae	Annona squamosa	0.0600	Fruit	Yes	456
Annonaceae	Annona cherimola	0.0260	Fruit	Yes	870
Arecaceae	Bactris gasipaes	0.4630	Fruit	Yes	170
Bignoniaceae	Tabebuia rosea	6.4360	Timber	Yes	673
Boraginaceae	Cordia alliodora	11.639	Timber	Yes	1474
Burseraceae	Bursera simaruba	4.7680	Timber	Yes	3316
Calophyllaceae	Mammea americana	0.0140	Fruit	Yes	112
Clusiaceae	Calophyllum brasiliense	0.1170	Timber	Yes	1386
Combretaceae	Terminalia oblonga	0.1770	Timber	Yes	380
Combretaceae	Terminalia amazonia	0.1240	Timber	Yes	715
Compositae	Perymenium grande	0.1980	Timber	Yes	258
Euphorbiaceae	Jatropha curcas	0.1530	N-fixing	Yes	683
Fagaceae	Quercus oleoides	0.1960	Timber	Yes	578
Juglandaceae	Juglans olanchana	0.1610	Timber	Yes	104
Lauraceae	Persea americana	1.0020	Fruit	Yes	1434
Leguminosae	Gliricidia sepium	12.326	N-fixing	Yes	864
Leguminosae	Erythrina costaricensis	2.7770	N-fixing	Yes	171
Leguminosae	Enterolobium cyclocarpum	2.0850	Timber	Yes	556
Leguminosae	Erythrina poeppigiana	1.5680	N-fixing	Yes	309
Leguminosae	Albizia saman	1.4360	Timber	Yes	359
Leguminosae	Inga vera	0.9600	N-fixing	Yes	2450
Leguminosae	Inga oerstediana	0.8430	N-fixing	Yes	526
Leguminosae	Albizia guachapele	0.6980	Timber	Yes	281
Leguminosae	Erythrina berteroana	0.4920	N-fixing	Yes	293
Leguminosae	Diphysa americana	0.4560	Timber	Yes	277
Leguminosae	Andira inermis	0.3920	Timber	Yes	808
Leguminosae	Cassia grandis	0.3920	Timber	Yes	381
Leguminosae	Inga punctata	0.3830	N-fixing	Yes	1242
Leguminosae	Schizolobium parahyba	0.2940	Timber	Yes	410
Leguminosae	Pentaclethra macroloba	0.2740	Timber	Yes	270
Leguminosae	Dalbergia glomerata	0.2640	Timber	Yes	149
Leguminosae	Acosmium panamense	0.2620	Timber	Yes	217
Leguminosae	Inga jinicuil	0.2020	N-fixing	Yes	168
Leguminosae	Hymenaea courbaril	0.1990	Timber	Yes	1228
Leguminosae	Leucaena leucocephala	0.1930	N-fixing	Yes	1518
Leguminosae	Inga micheliana	0.0880	N-fixing	Yes	82
Leguminosae	Platymiscium dimorphandrum	0.0880	Timber	Yes	84
Leguminosae	Inga ruiziana	0.0860	N-fixing	Yes	495
Leguminosae	Tamarindus indica	0.0860	Fruit	No	642
Leguminosae	Inga pavoniana	0.0780	N-fixing	Yes	224
Leguminosae	Inga cocleensis	0.0490	N-fixing	Yes	94

Continuation of Table S2					
Family	Species	Frequency	Main use	Neotropical	Presence locations
Leguminosae	Inga calderonii	0.0490	N-fixing	Yes	12865
Leguminosae	Inga densiflora	0.0490	N-fixing	Yes	10544
Leguminosae	Inga edulis	0.0490	N-fixing	Yes	10953
Leguminosae	Inga latibracteata	0.0490	N-fixing	Yes	96
Leguminosae	Inga laurina	0.0490	N-fixing	Yes	930
Leguminosae	Inga semialata	0.0490	N-fixing	Yes	11112
Leguminosae	Inga samanensis	0.0490	N-fixing	Yes	95
Leguminosae	Inga sapindoides	0.0490	N-fixing	Yes	11644
Leguminosae	Inga spectabilis	0.0490	N-fixing	Yes	11321
Leguminosae	Senna atomaria	0.0380	N-fixing	Yes	1530
Leguminosae	Acacia pennatula	0.0220	N-fixing	Yes	1375
Leguminosae	Acacia picachensis	0.0080	N-fixing	Yes	199
Leguminosae	Acacia hindsii	0.0070	N-fixing	Yes	287
Leguminosae	Erythrina lanata	0.0060	N-fixing	Yes	194
Leguminosae	Inga leiocalycina	0.0040	N-fixing	Yes	306
Leguminosae	Inga nobilis	0.0010	N-fixing	Yes	1011
Malpighiaceae	Byrsonima crassifolia	2.0990	Fruit	Yes	2152
Malvaceae	Guazuma ulmifolia	8.7530	Timber	Yes	5441
Malvaceae	Pachira quinata	2.5080	Timber	Yes	175
Malvaceae	Ceiba pentandra	0.1250	Timber	Yes	773
Malvaceae	Ochroma pyramidale	0.0530	Timber	Yes	632
Meliaceae	Cedrela odorata	3.9730	Timber	Yes	1734
Meliaceae	Swietenia macrophylla	0.9210	Timber	Yes	821
Meliaceae	Swietenia humilis	0.0920	Timber	Yes	411
Meliaceae	Carapa guianensis	0.0700	Timber	Yes	905
Moraceae	Maclura tinctoria	0.2990	Timber	Yes	1252
Moraceae	Brosimum alicastrum	0.2490	Fruit	Yes	1373
Moraceae	Artocarpus altilis	0.0130	Fruit	No	149
Myrtaceae	Psidium guajava	2.2520	Fruit	Yes	1465
Myrtaceae	Syzygium jambos	0.2490	Fruit	No	864
Myrtaceae	Pimenta dioica	0.1360	Fruit	Yes	492
Myrtaceae	Syzygium malaccense	0.0430	Fruit	No	163
Myrtaceae	Psidium friedrichsthalianum	0.0220	Fruit	Yes	68
Pinaceae	Pinus oocarpa	0.4600	Timber	Yes	789
Rubiaceae	Genipa americana	0.2390	Fruit	Yes	1318
Rubiaceae	Calycophyllum candidissimum	0.1090	Timber	Yes	477
Rutaceae	Citrus sinensis	1.6890	Fruit	No	257
Rutaceae	Citrus limon	0.2310	Fruit	No	299
Rutaceae	Citrus aurantiifolia	0.2050	Fruit	No	1330
Rutaceae	Citrus reticulata	0.0330	Fruit	No	152
Sapindaceae	Melicoccus bijugatus	0.0540	Fruit	Yes	145
Sapotaceae	Pouteria sapota	0.2870	Fruit	Yes	256
Sapotaceae	Chrysophyllum mexicanum	0.0750	Fruit	Yes	796
Sapotaceae	Chrysophyllum oliviforme	0.0490	Fruit	Yes	100
Sapotaceae	Chrysophyllum cainito	0.0360	Fruit	Yes	516
Sapotaceae	Manilkara zapota	0.0260	Fruit	Yes	633
Simaroubaceae	Simarouba glauca	0.1130	Timber	Yes	341
Urticaceae	Cecropia peltata	0.2070	Timber	Yes	830
Urticaceae	Cecropia obtusifolia	0.1070	Timber	Yes	572
End of Table S2					

Table S3. General circulation model (GCM) used to obtain climatic variables under scenarios RCP 4.5 and RCP 8.5 in 2050.

GCM	Abbreviation
ACCESS1-0	AC
BCC-CSM1-1	BC
CCSM4	CC
CNRM-CM5	CN
GFDL-CM3	GF
GISS-E2-R	GS
HadGEM2-AO	HD
HadGEM2-CC	HG
HadGEM2-ES	HE
INMCM4	IN
IPSL-CM5A-LR	IP
MIROC-ESM-CHEM	MI
MIROC-ESM	MR
MIROC5	MC
MPI-ESM-LR	MP
MRI-CGCM3	MG
NorESM1-M	NO

Table S4. Algorithms for environmental niche modelling included in the analysis of suitability of coffee, cocoa and tree species.

Algorithm	Method	Description
Envelope model	BIOCLIM	It computes the similarity of a location by comparing the fi (xi) at any location to a percentile distribution of the values at known locations of occurrence.
Multivariate distance	DOMAIN	It computes the Gower distance between environmental variables at any location and those at any of the known locations of occurrence.
Regression: Multivariate adaptive regression splines	MARS	It is a non-parametric regression technique that automatically models non-linearity and interactions between variables.
Flexible discriminant analysis	FDA	It is a supervise classification method. The method combines different models for multi-group non-linear classification.
Additive models: Generalized additive models	GAM	Semi-parametric approach to predicting non-linear responses to a suite of predictor.
Stepwise GAM	GAMSTEP	Builds a GAM model in a step-wise fashion.
Mixed GAM Computation Vehicle	MGCV	It provides functions for generalized additive and generalized additive mixed modelling.
Boosted regression models: Generalized boosted regression models	GBM	Based on prediction components, where each component consists of a different weighted sum of nonlinear transformations of the predictor variables.
Stepwise boosted regression tree models	GBMSTEP	It is a technique that aims to improve the performance of a single model by fitting many models based on stepwise selection and combining them for prediction.
Generalized linear models	GLM	Generalizes linear regression by allowing the linear model to be related to the response variable via a link function.
Stepwise generalized linear models	GLMSTEP	It includes regression models in which the predictive variables are selected by an automated algorithm that involves backward elimination or forward selection.
Maximum entropy	MAXENT	It is a machine-learning method that estimates the species distribution probability by assessing the maximum entropy distribution, so that the most spread-out, or closest to uniform.
Artificial neural networks	NNET	It is a machine learning approach that employs an adaptive structure, which can be trained with application data to capture complex relationships between input and out variables.
Random forests	RF	It is a collection of tree-structured weak learners that comprised identically distributed random vectors where each tree contributes to a prediction.
Recursive partitioning and regression trees	RPART	It is a simple nonparametric regression approach, where the space spanned by all predictor variables, is recursively partitioned into a set of rectangular areas. The partition is created such that observations with similar response values are grouped and a constant value of the response variable is predicted within each area.
Support vector machines	SVM	Machine-learning methods that are based on classification (C-svc, nu-svc), novelty detection (one-class-svc), and regression (eps-svr, nu-svr).
Support vector machines	SVME	It is used to train an SVM and carry out general regression and classification (of nu and epsilon-type), as well as density-estimation.
